# A Method for the Variational Calculation of Hyperfine-Resolved Rovibronic Spectra of Diatomic Molecules

**DOI:** 10.1021/acs.jctc.1c01244

**Published:** 2022-02-11

**Authors:** Qianwei Qu, Sergei N. Yurchenko, Jonathan Tennyson

**Affiliations:** Department of Physics and Astronomy, University College London, WC1E 6BT London, U.K.

## Abstract

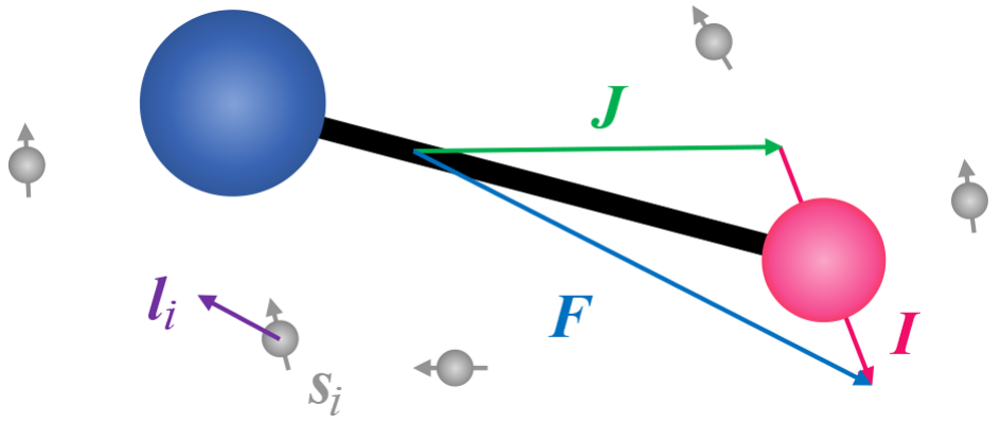

An algorithm for the calculation of hyperfine structure and spectra of diatomic molecules based on the variational nuclear motion is presented. The hyperfine coupling terms considered are Fermi-contact, nuclear spin-electron spin dipole–dipole, nuclear spin–orbit, nuclear spin-rotation, and nuclear electric quadrupole interactions. Initial hyperfine-unresolved wave functions are obtained for a given set of potential energy curves and associated couplings by a variation solution of the nuclear-motion Schrödinger equation. Fully hyperfine-resolved parity-conserved rovibronic Hamiltonian matrices for a given final angular momentum, ***F***, are constructed and then diagonalized to give hyperfine-resolved energies and wave functions. Electric transition dipole moment curves can then be used to generate a hyperfine-resolved line list by applying rigorous selection rules. The algorithm is implemented in Duo, which is a general program for calculating spectra of diatomic molecules. This approach is tested for NO and MgH, and the results are compared to experiment and shown to be consistent with those given by the well-used effective Hamiltonian code PGOPHER.

## 1 Introduction

The hyperfine structure of molecules lays the foundation for the studies of many important areas. The most immediate application is to reveal the properties of the molecules.^1−3^ Other examples include laser cooling experiments,^4,5^ astronomical observations,^6^ and, of course, nuclear magnetic resonance which has many applications including ones in medicine.

In the absence of external fields, the rotational hyperfine structure results from interactions between the electric and magnetic multipole moments of the nuclei and their molecular environments.^7^ Due to parity conservation inside the nuclei, only even electric and odd magnetic multipoles are nonvanishing. Although higher multipole effects are observed in some experiments, the dominant contributions to the hyperfine structure arise from magnetic dipole and electric quadrupole interactions.

Frosch and Foley^8^ performed a pioneering theoretical study of the magnetic interactions between nuclei and electron spins in diatomic molecules based on the Dirac equation, see discussion by Brown and Carrington.^9^ Bardeen and Townes^10^ provided the first extensive discussion of the electric quadrupole interactions.

The application of irreducible spherical tensor operators facilitates the evaluation of effective hyperfine Hamiltonian matrix elements,^7,9,11−13^ although one must still pay attention to anomalous commutation relationships when coupling angular momenta.^14,15^ Standard practice is to use these matrix elements to solve problems for which hyperfine structure is important using effective Hamiltonians which implicitly use a perturbation-theory-based representation of the problem.^3,6^ The effective Hamiltonian of a fine or hyperfine problem is usually constructed within a particular vibrational state, and the rotational coupling terms are treated as perturbations. The assumptions implicit in this approach are usually valid because the splitting of the (rotational) energy levels due to hyperfine effects are generally small compared to the separation between electronic or vibrational states. However, this assumption can fail, such as for example, for Rydberg states of molecules.^16,17^ The B ^2^Π–C ^2^Π avoided crossing structure in NO is another example of strong electronic state interaction. The perturbative treatment of this vibronic coupling is difficult: it requires a lot of parameters,^18^ and is not very accurate. The interaction between different states leads to significant complications which are difficult to model using the standard effective Hamiltonian approach.

In contrast, our recent work on a spectroscopic model for the four lowest electronic states of NO^19^ proposed a compact solution for the problem based on the use of a variational method to treat the nuclear motion. In our approach, which is based on the use of potential energy curves and appropriate couplings, it was only necessary to introduce one potential energy coupling curve between the coupled B ^2^Π and C ^2^Π electronic states; this gave an accurate rovibronic line list for NO.^20^ These calculations used a general program for the calculation of spectra of diatomic molecules, Duo.^21^

Duo is a variational nuclear motion program developed for the calculation of rovibronic spectra of diatomic molecules as part of the ExoMol project.^22^ It provides explicit treatment of spin–orbit and other coupling terms and can generate high-accuracy fine-structure diatomic line lists. Duo has been used to generate many line lists including those for AlO,^23^ CaO,^24^ VO,^25^ TiO,^26^ YO,^27^ and SiO,^28^ which are provided via the ExoMol database.^29^Duo was also recently employed to calculate temperature-dependent photodissociation cross sections and rates.^30^Duo has also been adapted to treat ultralow energy collisions as the inner region in an R-matrix formalism;^31^ hyperfine effects are very important in such collisions. Recently, a new module treating electric quadrupole transitions has been added to Duo,^32^ which makes it capable of predicting spectra for diatomic molecules with no electric dipole moment, for example O_2_ and N_2_. However, up until now Duo had not treated hyperfine effects. In this context we note that hyperfine coupling is particularly strong for VO,^33,34^ meaning that the current ExoMol VO line list, VOMYT^25^ which is not hyperfine resolved, is unsuitable for high resolution work, such as the study of exoplanets using high-resolution Doppler-shift spectroscopy.^35^

Here we present a variational procedure for calculating hyperfine-resolved spectra of diatomic molecules. The new algorithm we design is implemented as new modules in Duo. In general, the most challenging part of solving quantum mechanical problems using a variational method is finding good variational basis sets. We show below that Duo gives appropriate basis sets thanks to its well-designed calculation hierarchy and algorithm. Numerical tests indicates that the algorithm proposed here can achieve high accuracy for the calculation of hyperfine structure.

## 2 Overview

**Figure 1:**
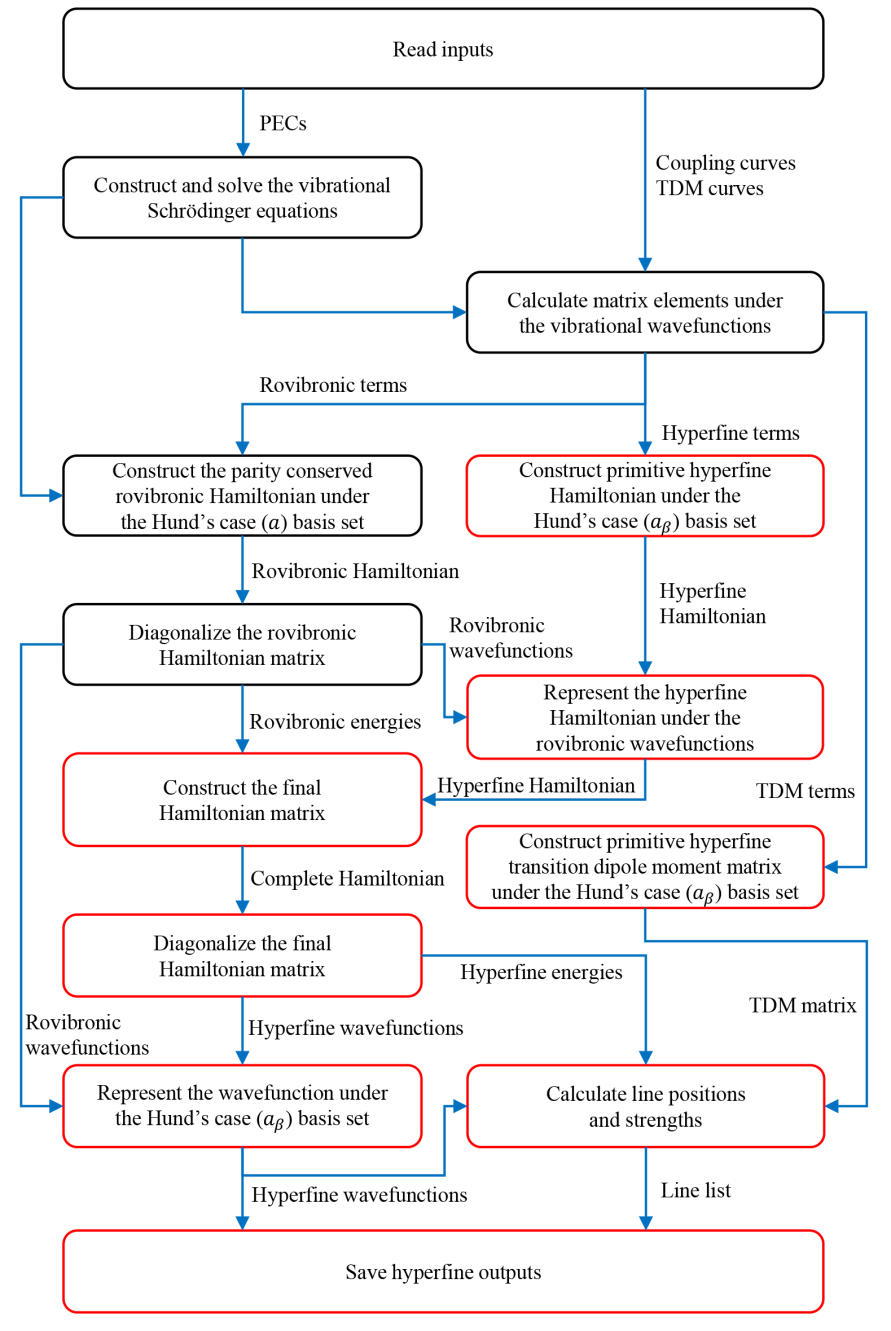
Flowchart showing the structure of a Duo hyperfine calculation. Existing modules are given by black rectangles while new modules are denoted by red rectangles. PEC is short for potential energy curve and TDM is short for transition dipole moment.

In this section, we outline our algorithm so that the readers can easily follow the details given in the following sections. Figure 1 gives a graphical representation of the algorithm.

We write the Hamiltonian for the problem as
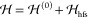
1where  is the rovibronic Hamiltonian which Duo originally used to give fine structure resolved solutions for diatomic molecules, and  gives the nuclear hyperfine interaction terms introduced in this work. We emphasize that although this structure is the standard one used in perturbation theory, here we aim for a full variational solution of the whole Hamiltonian .

### 2.1 Rovibronic Fine Structure

Duo has well-developed modules, surrounded by black rectangles in Figure 1, for the calculation of rovibronic energies and wave functions.

The computational procedure used by Duo to obtain solutions for  is divided into two steps. First, the rotationless Schrödinger equation is solved independently for each uncoupled potential energy curve, *V*_state_(*R*), to give vibrational energy levels, *E*_state,*v*_, and wave functions, ψ_state,*v*_:
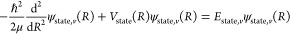
2where *R* is the internuclear distance, μ is the reduced mass of the molecule, “state ” and *v* indicate the electronic state and vibrational quantum numbers. Duo employs contracted vibrational basis sets given by ψ_state,*v*_ = |state, *v*⟩ to define a finite-dimension space.

In the second step, a rovibronic Hamiltonian matrix, corresponding to , for each specific total angular momentum exclusive of nuclear spin, *J*, and parity, τ, is constructed using a Hund’s case (a) basis set:^36^
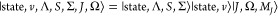
3which is decoupled into three parts: (i) the electronic eigenfunction, (ii) the vibrational eigenfunction of eq 2, and (iii) the rotational eigenfunction of a symmetric top. The quantum numbers in eq 3, state, *v*, Λ, *S*, Σ, J, Ω, and *M*_*J*_, correspond to the electronic state, the vibrational eigenstate, the projection of the electron orbital angular momentum ***L*** on the molecular axis, the projection of the electron spin angular momentum ***S*** on the molecular axis, the projection of ***J*** on the molecular axis, and the projection of ***J*** on the space-fixed *Z*-axis, respectively. Note that, Duo calculates the spectra of diatomic molecules in field-free environments. Thus, we do not really use *M*_*J*_ to construct the basis set, as the left-hand side of eq 3 indicates. All the angular momenta are quantized to the body-fixed axes.

When evaluating the matrix elements using the basis functions of eq 3, the necessary coupling curves are integrated over pairs of vibrational basis functions:
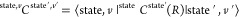
4where *C*(*R*) can be either a diagonal coupling curve for a particular electronic state or an off-diagonal coupling curve between two states. Supported couplings include electron spin–orbit, electron spin–spin, electron spin–rotation *etc*.^21,36^

The basis functions of eq 3 do not have definite parities. Duo uses linear combinations of them to define parity-conserved basis functions:
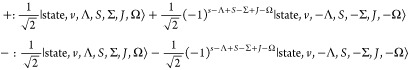
5where *s* = 1 for Σ^–^ states and *s* = 0 for all other states. Note that, the parity is independent of *M*_*J*_. Each matrix of  constructed using these basis functions can be diagonalized to give rovibronic energy levels and wave functions of a definite *J* and parity τ. Let |ϕ_*m*_^τ,*J*^⟩ be the *m*th eigenfunction corresponding to a given *J* and parity τ, we have
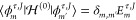
6where *E*_*m*_^τ,*J*^ is the *m*th eigenvalue.

Thanks to the use of complete angular basis sets and the variational method, the final energies are independent of the coupling scheme used. If there is enough vibrational basis (determined by the users’ setup), the choice of Hund’s case (a) will give correct results even for cases for which other coupling schemes provide a better zeroth-order approximation.

### 2.2 Nuclear Hyperfine Structure

We program new Duo modules to accomplish the functions denoted by the red rectangles in Figure 1 for nuclear hyperfine structure calculations. We only consider heteronuclear diatomic molecules with one nucleus possessing nonzero spin in this paper. In this case, nuclear spin, ***I***, is coupled with ***J*** to give total angular momentum, ***F***, that is,
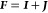
7To evaluate the matrix elements of , we introduce the following primitive basis functions
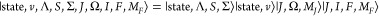
8where the angular momenta ***I*** and ***F*** are quantized to the space-fixed axes; ***J*** is quantized to both the space-fixed and the body-fixed axes; ***L*** and ***S*** are quantized to the body-fixed axes. Without an external field, *M*_*F*_ can be omitted:
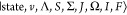
9The basis functions are countable in Duo and thus, can be simply denoted as
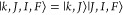
10where *k* is a counting number for the basis functions associated with a given *J*. It is an equivalent representation of eq 9 and |*k*, *J*⟩ is short for eq 3.

**Figure 2:**
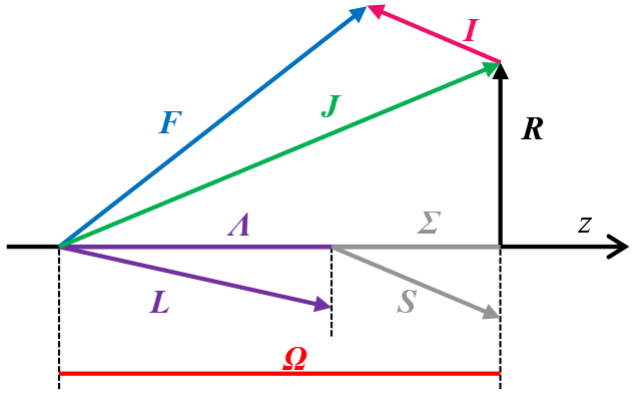
Hund’s case (a_β_) angular momenta coupling scheme. ***R*** is the rotational angular momentum of bare nuclei.

The quantum numbers, *J*, *I*, and *F*, satisfy the triangle inequality:
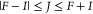
11The coupling scheme used is known as Hund’s case (a_β_),^8^ and is illustrated in Figure 2. We emphasize that because we use complete angular basis sets, our results are independent of the coupling scheme used and its choice largely becomes one of algorithmic convenience.

To obtain a parity-conserved basis set, we rely on the symmetrization procedure given in eq 5 by making use of the eigenfunctions obtained as solutions of , |ϕ_*m*_^τ,*J*^⟩, to define the basis functions:
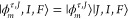
12The parity conserved rovibronic basis functions, eq 12, can be represented by the primitive basis functions, eq 9 or eq 10
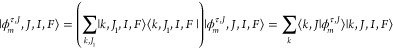
13where the coefficients, ⟨*k*, *J*|ϕ_*m*_^τ,*J*^⟩, have been obtained when calculating rovibronic fine structure by solving for . The matrix elements of  in this basis functions are straightforward
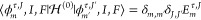
14Therefore, constructing the hyperfine-resolved matrix elements
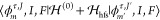
just requires the matrix elements of , .

In practice, we first construct the matrix elements of  using the primitive basis functions of eq 9 and then transform to the representation of  of eq 12 using a basis transformation. The mathematical and physical details are discussed in the next two sections. Before that, we outline the algorithm used to calculate hyperfine-resolved spectra.

As a first step, the hyperfine coupling curves, such as the Fermi contact interaction curves,^37^ are integrated over the vibrational wave functions. Duo uses these vibrational matrix elements to compute the hyperfine matrix elements within a Hund’s case (a_β_) basis set, eq 9, and constructs a Hamiltonian matrix for each specific total angular momentum, *F*. Next, the matrix, corresponding to  is constructed in the representation of eq 12. After this step, the hyperfine matrix elements are parity conserved. Combining the rovibronic energies and hyperfine matrix elements, Duo constructs the complete Hamiltonian matrix, corresponding to , for each given value of *F* and τ. Diagonalizing this matrix gives the hyperfine-resolved energy levels and corresponding wave functions in the representation of eq 12. Finally, the eigenfunctions are transformed back to Hund’s case (a_β_) representation of eq 9 as this representation is more convenient to use for hyperfine-resolved intensity calculations, for analysis of wave functions, and to assign quantum numbers to hyperfine states.

## 3 The Hyperfine Structure Hamiltonian

We investigate the field-free hyperfine structure of diatomic molecules in which only one of the nuclei possesses nuclear spin, and consider five nuclear hyperfine terms in this work:
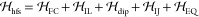
15They are, respectively, the Hamiltonians of the Fermi contact interaction, the nuclear spin–orbit interaction, the nuclear spin–electron spin dipole–dipole interaction, the nuclear spin–rotation interaction, and the nuclear electric quadrupole interaction. These Hamiltonians have the following definitions:^9,13^
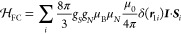
16
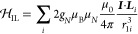
17
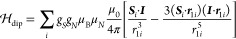
18
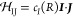
19
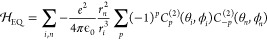
20

The constants, *e*, *g*_*S*_, μ_B_, *g*_*N*_, μ_*N*_, and μ_0_, are the elementary charge, the free electron spin *g*-factor, the electron Bohr magneton, the nuclear spin *g*-factor, the nuclear magneton, and the vacuum permeability, respectively. ***I*** is the spin of the nucleus of interest (defined as nucleus 1), ***r***_1*i*_ is the relative position between the *i*th electron and nucleus 1, ****S***_i_* is the spin of the *i*th electron, ***L***_*i*_ is the orbit angular momentum of the *i*th electron, and δ(·) is the Dirac delta function. In eq 19, we introduce the nuclear spin-rotation interaction constant, *c*_*I*_(*R*), which is a function of internuclear distance. Section 8.2.2(d) of Brown and Carrington^9^ and Miani and Tennyson^38^ define the nuclear spin-rotation tensor and how it can be reduced to a constant for a diatomic molecule. In eq 20, *C*_*p*_^(2)^ is the modified rank-2 spherical harmonic:
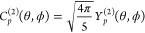
21where *Y*_*p*_^(2)^(θ, ϕ) is the standard spherical harmonic; (*r*_*i*_, θ_*i*_, ϕ_*i*_) and (*r*_*n*_, θ_*n*_, ϕ_*n*_) are the positions of the *i*th electron and the *n*th proton, respectively.

The first four hyperfine Hamiltonians, given by eqs 16–19, are nuclear magnetic dipole terms resulting from the interactions between the magnetic dipole moment given by nuclear spin and magnetic fields due to the motion of nuclei or electrons. The nuclear electric quadrupole Hamiltonian arises from the interaction between the nuclear electric quadrupole moment and the electric field inside a molecule. The nuclear spin-rotation interaction is usually much weaker than the other four hyperfine terms (if nonzero). See Table 1 of Broyer et al.^12^ for the order of magnitude of the hyperfine terms.

To aid the evaluation of matrix elements, the hyperfine Hamiltonians can be written as scalar products of irreducible tensor operators:^9^
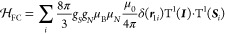
22
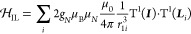
23
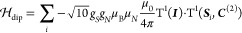
24
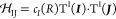
25
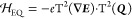
26where T^*k*^(·) indicates a rank-*k* tensor. All the tensors here are defined in space-fixed frame. The two tensors in eq 26 defining the gradient of electric field and the nuclear quadrupole moment are, respectively:
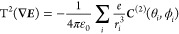
27
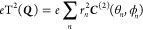
28

## 4 Matrix Elements of the Hyperfine Structure

### 4.1 Primitive Matrix Elements of the Hyperfine Structure

In this section, primitive matrix elements of the hyperfine structure are initially evaluated in the representation of eq 9. In this work, we do not consider hyperfine couplings between different electronic states when evaluating primitive matrix elements, which are, thus, diagonal in the electronic state and electron spin, that is, 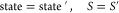
in the bra-ket notation, and immediately we have
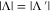


As ***F*** = ***J*** + ***I***, we can initially decouple the representation of |*J*, *I*, *F*, *M*_*F*_⟩ in eq 8 to uncoupled ones; see Edmonds^39^ for a formal definition and irreducible spherical tensor operators. Taking the Fermi contact term as an example, the nonvanishing matrix element on the primitive basis functions for *M*_*F*_ = *M*′_*F*_ is
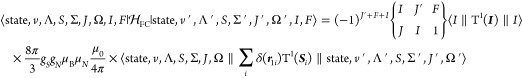
29where  is the Wigner-6*j* symbol.

The nuclear spin is quantized to the space-fixed axes, and thus, the reduced matrix element of T^1^(***I***) is
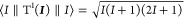
30The electron spin is quantized to the body-fixed axes. To evaluate the second reduced matrix element in eq 29, the electron spin spherical tensor is rotated from the space-fixed frame to the body-fixed frame in which the components of the tensors are denoted by *q*:
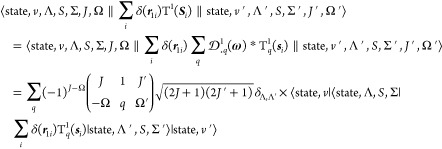
31where ***s***_i_ is the spin of the *i*th electron in body-fixed system,  is a Wigner rotation matrix and  is a Wigner-3*j* symbol. The electron tensor operators, T_*q*_^1^(***s***_*i*_), do not directly act on the electronic part of Hund’s case (a) basis. We may replace the electron spin operators with an effective one:
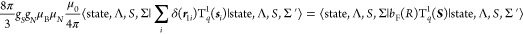
32where ***S*** is the total spin. Requiring Σ = Σ^′^, the Fermi contact interaction curve can be defined as^37^
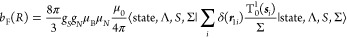
33where T_0_^1^(***s***_*i*_)/Σ represents the projection operator for each electron *i* (see eq (7.152) of Brown and Carrington^9^). On the basis of eqs 29 to 33, we finally get
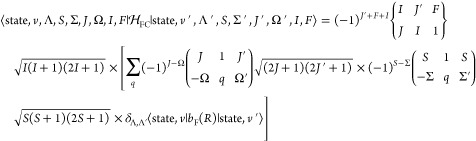
34Other hyperfine matrix elements can be evaluated analogously.

For the nuclear spin–orbit term, we are only interested in the diagonal matrix elements of Λ
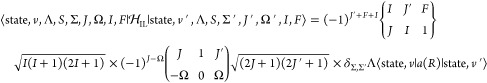
35The nondiagonal couplings between different electronic states via T_±1_^1^(***L***) are not considered here. The diagonal nuclear spin–orbit interaction curve is defined as^37^
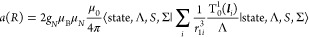
36where ***l***_i_ is the orbital angular momentum of the *i*th electron defined in the body-fixed frame.

The nuclear spin-electron spin dipole–dipole interaction is somewhat complicated. With the definition (see Appendix 8.2 of Brown and Carrington^9^)
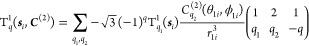
37where (*r*_1*i*_, θ_1,*i*_, ϕ_1,*i*_) are the spherical polar coordinates of electron *i* relative to nucleus 1, we shall give two kinds of matrix elements. For the term diagonal in Λ, that is, *q*_2_ = 0 and *q* = *q*_1_:
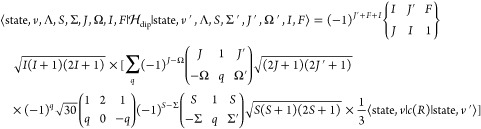
38The diagonal nuclear spin-electron spin dipole–dipole interaction constant curve is defined as,^37^
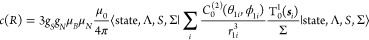
39For the off-diagonal terms of  in Λ and Λ′ which satisfy *q*_2_ = ∓2, that is, *q*_1_ = ±1 and *q* = ∓1, we have
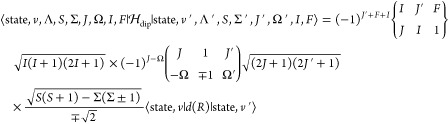
40The off-diagonal nuclear spin-electron spin dipole–dipole interaction constant curve is defined as,^37^
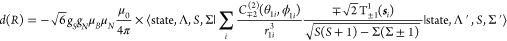
41

The case of the nuclear spin-rotation interaction is much simpler, as it is not necessary to rotate T^1^(***J***) to the body-fixed axis system:
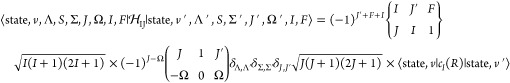
42To evaluate the matrix elements for the electric quadrupole interaction, we decouple the inner product of second rank irreducible tensors:
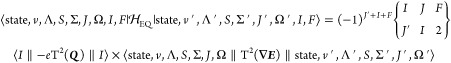
43The electric quadrupole reduced matrix element is nonzero only if *I* ≥ 1; it can be evaluated as
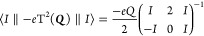
44where *eQ* is the nuclear electric quadrupole moment; see Cook and De Lucia^7^ or Appendix 8.4 of Brown and Carrington.^9^ The values of *Q* for various atoms were collected by Pyykkö.^40^ The reduced matrix element of the gradient of electric field is
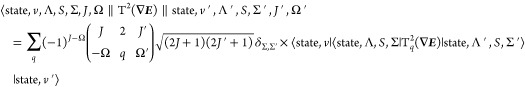
45The diagonal and off-diagonal *R*-dependent constants of the gradient of electric field are respectively defined as (see eqs (7.159) and (7.163) of Brown and Carrington^9^):
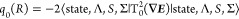
46
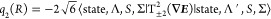
47Note that sometimes *q*_0_ is denoted as *q*_1_, see for example, eq (2.3.76a) of Hirota.^41^ We follow the convention of Brown and Carrington^9^ and preserve the variable *q*_1_ for the nuclear electric quadrupole coupling constant between different electronic states arising from T_±1_^2^(***∇E***) which will be the subject of future work. Finally, the diagonal matrix elements of nuclear electric quadrupole coupling are
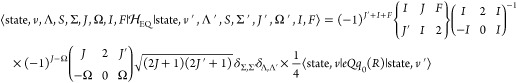
48while the off-diagonal ones are
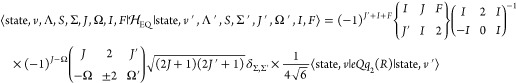
49As we only consider the hyperfine interactions within a particular electronic state in this paper, the off-diagonal matrix elements arising from *d*(*R*) in eq 41 and *q*_2_(*R*) in eq 47 only contribute to the Λ-doubling terms of Π states. In the electron spin resonance spectroscopy literature, the Fermi-contact and nuclear spin–electron spin dipole–dipole terms are, respectively, the first-order isotropic and dipolar contributions to the hyperfine coupling **A**-tensor.^42^ When the second-order contributions of paramagnetic spin orbit (PSO) interaction are considered, the hyperfine coupling constants defined in this paper can be further revised by the PSO terms and determined by the matrix elements of the total hyperfine **A**-tensor.^43^

### 4.2 Parity Conserved Matrix Elements under the Rovibronic Wave Functions

Recall the short notation of Hund’s case (a_β_) basis in eq 10, |*k*, *J*, *I*, *F*⟩ and the basis functions we defined in eq 12, |ϕ_*m*_^τ,*J*^, *I*, *F*⟩; the hyperfine matrix elements under the basis set can be expanded as
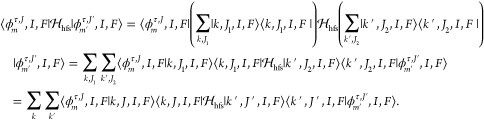
50We can rewrite the basis transformation into the matrix format:
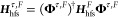
51,  and ⟨*k*′, *J*′, *I*, *F*|ϕ_*m*′_^τ,*J*′^, *I*, *F*⟩ are the matrix elements of ***H***_hfs_^τ,F^, ***H***_hfs_^*F*^, and **Φ**^τ, *F*^, respectively, and,
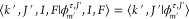


### 4.3 Solution for the Hyperfine Structure

The final Hamiltonian which is constructed from summation of the rovibronic and hyperfine matrices
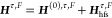
52where ***H***^(0),τ,*F*^ is the matrix of  (see eq 14 for the matrix elements). Diagonalizing the parity-conserved matrix of each *F* results in the energies and wave functions of the hyperfine structure:
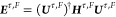
53The eigenfunction matrix ***U***^τ,*F*^ is represented in the parity-conserved rovibronic basis set defined in eq 12, which is, however, not very useful for quantum number assignments and wave function analysis. For these purposes, the wave functions can be transformed back in the representation of Hund’s case (a) basis set and the final wave function matrix is
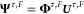
54Here, we denote the countable rovibronic wave functions considering nuclear hyperfine interaction as
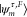
55such that
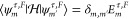
56where *E*_*m*_^τ,*F*^ is the corresponding eigenvalue of |ψ_*m*_^τ,*F*^⟩.

The basis transformation procedures from eq 50 to eq 54 reveal the key feature of our variational method which involves accounting for the contribution of every basis function to the final eigenstates. Finally, only *F*, τ, and counting number *m* are good quantum numbers.

## 5 Line Strength of the Hyperfine Transitions

In the absence of an external field, the line strength of a nuclear spin resolved rovibronic transition is defined by^7^
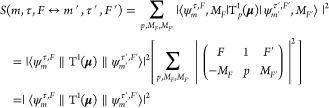
57We initially evaluate the reduced matrix elements of the electric dipole moment in the representation of eq 9 and then calculate the reduced line strength matrix elements by matrix multiplication:
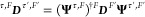
58where ^*F*^***D***^*F*′^ and ^τ, *F*^***D***^τ′, *F*′^ are the reduced transition dipole moment matrices in the representation of eq 9 and eq 55, respectively. The following equations give the elements of ^*F*^***D***^*F*′^, that is, ⟨*k*, *J*, *I*, *F*∥T^1^(**μ**)∥*k*′, *J*′, *I*, *F*⟩.

As ***F*** = ***J*** + ***I*** and T^1^(**μ**) commutes with ***I***,
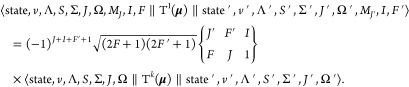
59Rotating the spherical tensor to the body-fixed frame gives
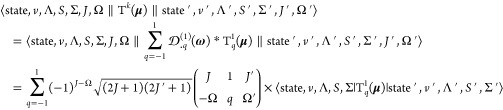
60The matrix element ⟨state, *v*, Λ, *S*, Σ|T_*q*_^1^(**μ**)|state′, *v*′, Λ′, *S*′, Σ′⟩ is the same as the one used for the calculation of rovibronic transition intensities excluding nuclear spin in Duo,^21^
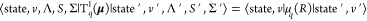
61where μ_*q*_(*R*) is the electric dipole moment curve represented in the body-fixed frame which can be obtained from *ab initio* calculation.

For dipole moment transitions, parity has to be changed and thus follows the selection rule:
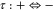
62The selection rules on *F* comes from the Wigner-6*j* symbol of eq 59:
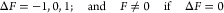
63The hyperfine Hamiltonian mixes wave functions with different *J*; as a result, electric dipole transition “forbidden” lines with |*ΔJ*| > 1 are observable. For example, when *I* = ^1^/_2_, we can observe electric dipole transitions of *O* and *S* branches (*ΔJ* = ±2), even if they might be much weaker than the transitions of *P*, *Q*, and *R* branches.

## 6 Numerical Verification

To illustrate and validate our new hyperfine modules, we calculate hyperfine-resolved rotational spectra for electronic and vibrational ground state of ^14^N^16^O and ^24^Mg^1^H. While both ^16^O and ^24^Mg have nuclear spin zero; ^14^N has *I* = 1 and ^1^H has *I* = ^1^/_2_ which allows us to test different coupling mechanisms. For this purpose we compare the results of our Duo calculations with that of PGOPHER^44^ using the same model for each calculation. PGOPHER obtains the energy levels and spectra from effective Hamiltonians given appropriate spectral constants. In contrast, Duo takes in coupling curves and performs variational calculations. To get consistent inputs between the two codes it was necessary to simplify the treatment used by Duo.

For ^14^N^16^O we approximate the Duo solution by using only one contracted vibrational basis function, that is; |X ^2^Π, *v* = 0⟩ which ensures that we avoid any hyperfine-induced interaction between different vibrational states. In PGOPHER, we used values for the rotational constant, *B*_0_, and spin–orbit coupling constant matrix, *A*_0_, computed using Duo:
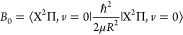
64
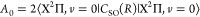
65where μ is the reduced mass of ^14^N^16^O and *C*_SO_(*R*) is the spin–orbit coupling curve. Note that, for spin–orbit interaction, the coupling curve, *C*_SO_(*R*), describes the coupling energies, while the constant, *A*, is defined by the splitting energies. Thus, *A* is defined by twice the matrix element. The NO X ^2^Π potential energy curve used by Duo was taken from Wong et al.^45^*C*_SO_(*R*) was assigned an artificial constant *C*_SO_(R) = 60 cm^–1^ and the transition dipole moment curve was set to 1 D. Our adopted values for *B*_0_ and *A*_0_ are given in Table 1.

**Table 1:** Spectroscopic Constants for ^14^N^16^O Used in This Paper

constants	values [cm^–1^]
*B*_0_	1.696 084 011 913 95
*A*_0_	120

For this analysis, the hyperfine coupling was chosen using artificial curves much greater than experimental values. By including only one hyperfine constant at a time, we test the affects of a particular hyperfine interaction. The results are compared in Table 2. Note that, PGOPHER uses nuclear spin-electron spin constants, *b*, defined by Frosch and Foley,^8^ rather than *b*_F_. They are related by the dipole–dipole constant, *c*,
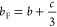
66Duo achieves excellent agreement with PGOPHER for the calculation of both the line positions ν and line strengths *S*. The slight differences are due to rounding error. As we did not include Λ-doubling terms in our calculation the wavenumbers corresponding to *b*_F_, *a*, *eQq*_0_, and *c*_*I*_ in the first and second columns of the same *F* = 0.5 (or in the third and fourth columns, *F* = 1.5) of Table 2 are the same. Hyperfine interactions only split the transitions of different *F* in the first and third columns (or in the second and fourth columns). In contrast, the wavenumbers obtained with *eQq*_2_ or *d* included are different from each other even for the same values of *F* due to the hyperfine contribution to both Λ-doubling and hyperfine splitting.

**Table 2:** Comparison of ^14^N^16^O Line Positions and Line Strengths for Calculated Results from Duo and PGOPHER^a^

Number		1	2	3	4
upper	*F*′	0.5	0.5	1.5	1.5
	τ″	–	+	–	+
	*J*″	1.5	1.5	1.5	1.5
lower	*F*″	0.5	0.5	0.5	0.5
	τ″	+	–	+	–
	*J*″	0.5	0.5	0.5	0.5
*b* = 0.1 *c* = 0.3	νDuo	148343.21846	148343.21846	147225.55589	147225.55589
	ν_PG_	148343.21850	148343.21850	147225.55590	147225.55590
	*S*Duo	0.60757296	0.60757296	0.77125182	0.77125182
	*S*_PG_	0.60757300	0.60757300	0.77125180	0.77125180
*a* = 0.1	νDuo	151349.03162	151349.03162	151956.77196	151956.77196
	ν_PG_	151349.03160	151349.03160	151956.77200	151956.77200
	*S*Duo	0.58421238	0.58421238	0.72433238	0.72433238
	*S*_PG_	0.58421240	0.58421240	0.72433240	0.72433240
*eQq*_0_ = 0.1	νDuo	149591.09156	149591.09156	150930.88155	150930.88155
	ν_PG_	149591.09160	149591.09160	150930.88160	150930.88160
	*S*Duo	0.59805081	0.59805081	0.73432902	0.73432902
	*S*_PG_	0.59805080	0.59805080	0.73432900	0.73432900
*c*_*I*_ = 0.1	νDuo	145827.72503	145827.72503	150324.61190	150324.61190
	ν_PG_	145827.72500	145827.72500	150324.61190	150324.61190
	*S*Duo	0.59221720	0.59221720	0.74027149	0.74027149
	*S*_PG_	0.59221720	0.59221720	0.74027150	0.74027150
*eQq*_2_ = 0.1	νDuo	150346.43930	150302.88914	150307.21201	150342.05212
	ν_PG_	150346.43930	150302.88910	150307.21200	150342.05210
	*S*Duo	0.59221687	0.59221668	0.74027121	0.74027140
	*S*_PG_	0.59221690	0.59221670	0.74027120	0.74027140
*d* = 0.1	νDuo	150329.98859	150332.52077	149133.39987	151532.62042
	ν_PG_	150329.98860	150332.52080	149133.39990	151532.62040
	*S*Duo	0.59210956	0.59211520	0.75214574	0.72851989
	*S*_PG_	0.59210960	0.59211520	0.75214570	0.72851990

a Hyperfine constants are in cm^–1^ and line positions are given in MHz. The line strength, *S* [Debye^2^], has the same definition as that in PGOPHER when the intensity unit option of PGOPHER, IntensityUnit, is chosen as HonlLondon and the transition dipole moment is set to 1 D.

We also tested the code for an *I* = ^1^/_2_ case by calculating pure rotational transitions within the *v* = 0, X ^2^Σ^+^ state of ^24^MgH, again using a unit electric dipole moment curve. This is a rather realistic case, as the input spectral constants to PGOPHER listed in Table 3 were determined by the observed transitions.^46^ As for the input to Duo, the potential energy curve was shifted from an empirically determined one^47,48^ to reproduce the *B*_0_ constant given in Table 3, that is;
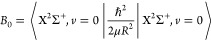
67The curves of spin-rotation and hyperfine couplings were defined as
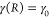
68
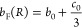
69
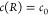
70Note that the contribution of *D*_0_ is not allowed for when only one contracted basis function is used in Duo. Just like the *B*_*v*_ constant, Duo does not use rotational constants, *D*_*v*_, *H*_*v*_, etc., either, and introduction of these centrifugal distortion would require manipulation of the potential energy curves which are beyond the scope of this work. Nevertheless, Duo still gives hyperfine splittings which are consistent with PGOPHER, see the comparison in Table 4, because *D*_0_ uniformly shifts the hyperfine energy levels within the same *N* rotational levels, where *N* is the quantum number corresponding to ***N*** which is defined as
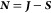
71

**Table 3:** X ^2^Σ^+^, *v* = 0 Spectral Constants of ^24^Mg^1^H Determined by Ziurys et al.^46^ These Values Were Used as the Input to PGOPHER

constants	values [MHz]
*B*_0_	171976.1782
*D*_0_	10.6212
γ_0_	790.809
*b*_0_	306.277
*c*_0_	4.792

**Table 4:** Comparison of ^24^Mg^1^H X ^2^Σ^+^, *v* = 0 Hyperfine Energies Calculated by Duo and PGOPHER^a^

no.	*F*	τ	*J*	*N*	*E*Duo	*E*_PG_	difference
1	0	+	0.5	0	–230.9057	–230.9057	0.0000
2	1	+	0.5	0	76.9686	76.9686	0.0000
3	1	–	0.5	1	343117.2196	343074.7347	42.4849
4	0	–	0.5	1	343236.9188	343194.4339	42.4849
5	1	–	1.5	1	344238.9505	344196.4655	42.4850
6	2	–	1.5	1	344424.5699	344382.0849	42.4850

a Only one vibrational contracted basis function |X ^2^Σ^+^, *v* = 0⟩ was used in this case. All energies are given in MHz.

We then allowed for the effect of vibrational coupling in Duo by increasing the contracted vibration bases to five functions, that is, |X ^2^Σ^+^, v = 0, 1, 2, 3, 4⟩. As shown in Table 5, vibrational coupling from higher vibrational states automatically introduces centrifugal distortion to the *v* = 0 state and improves the accuracy of the calculation, compared with the lower rotational levels in Table 4. We did not use a very accurate model here, and thus for higher rotational levels, we still got obvious energy differences in Table 5, and frequency differences in Table 6. The best way to achieve experimental accuracy is to refine the curves by fitting calculated energies or frequencies to measured ones.

**Table 5:** Comparison of ^24^Mg^1^H X ^2^Σ^+^, *v* = 0 Hyperfine Energies Calculated by Duo and PGOPHER^a^

no.	*F*	τ	*J*	*N*	*E*Duo	*E*_PG_	difference
1	0	+	0.5	0	–230.9058	–230.9057	–0.0001
2	1	+	0.5	0	76.9686	76.9686	0.0000
3	1	–	0.5	1	343074.6047	343074.7347	–0.1300
4	0	–	0.5	1	343194.3039	343194.4339	–0.1300
5	1	–	1.5	1	344196.3356	344196.4655	–0.1299
6	2	–	1.5	1	344381.9550	344382.0849	–0.1299
7	2	+	1.5	2	1030229.8178	1030230.9249	–1.1071
8	1	+	1.5	2	1030363.5553	1030364.6624	–1.1071
9	2	+	2.5	2	1032168.8370	1032169.9441	–1.1071
10	3	+	2.5	2	1032341.1483	1032342.2554	–1.1071
11	3	–	2.5	3	2060535.9577	2060540.0064	–4.0487
12	2	–	2.5	3	2060675.3485	2060679.3973	–4.0488
13	3	–	3.5	3	2063276.7730	2063280.8218	–4.0488
14	4	–	3.5	3	2063443.5527	2063447.6015	–4.0488
15	4	+	3.5	4	3433222.1380	3433231.9781	–9.8401
16	3	+	3.5	4	3433364.6194	3433374.4596	–9.8402
17	4	+	4.5	4	3436759.8067	3436769.6469	–9.8402
18	5	+	4.5	4	3436923.5400	3436933.3802	–9.8402
19	5	–	4.5	5	5147267.6517	5147285.8407	–18.1890
20	4	–	4.5	5	5147412.0861	5147430.2751	–18.1890
21	5	–	5.5	5	5151599.9592	5151618.1483	–18.1891
22	6	–	5.5	5	5151761.7609	5151779.9499	–18.1890
23	6	+	5.5	6	7201400.1636	7201426.5351	–26.3715
24	5	+	5.5	6	7201545.9449	7201572.3164	–26.3715
25	6	+	6.5	6	7206525.9256	7206552.2971	–26.3715
26	7	+	6.5	6	7206686.3922	7206712.7637	–26.3715
27	7	-	6.5	7	9594096.6941	9594124.3704	–27.6763
28	6	-	6.5	7	9594243.4608	9594271.1371	–27.6763
29	7	-	7.5	7	9600015.2023	9600042.8786	–27.6763
30	8	-	7.5	7	9600174.6909	9600202.3672	–27.6763
31	8	+	7.5	8	12323585.3054	12323594.8594	–9.5540
32	7	+	7.5	8	12323732.8245	12323742.3785	–9.5540
33	8	+	8.5	8	12330296.1028	12330305.6568	–9.5540
34	9	+	8.5	8	12330454.8439	12330464.3979	–9.5540
35	9	-	8.5	9	15387847.1770	15387798.6594	48.5176
36	8	-	8.5	9	15387995.2894	15387946.7718	48.5176
37	9	-	9.5	9	15395349.9512	15395301.4336	48.5176
38	10	-	9.5	9	15395508.1024	15395459.5848	48.5176

a Five vibrational contracted basis functions |X ^2^Σ^+^, *v* = 0, 1, 2, 3, 4⟩ were used in this case. All energies are given in MHz.

**Table 6:** Comparison of ^24^Mg^1^H X ^2^Σ^+^, *v* = 0 Hyperfine Line Positions^a^

no.	*N*′	*J*′	*F*′	*N*″	*J*″	*F*″	νDuo	measured (a)^46^	measured (b)^49^
1	1	0.5	1	0	0.5	1	342997.636	342997.763(050)	
2	1	0.5	0	0	0.5	1	343117.335	343117.463(050)	
3	1	0.5	1	0	0.5	0	343305.510	343305.646(050)	
4	1	1.5	1	0	0.5	1	344119.367	344119.497(050)	
5	1	1.5	2	0	0.5	1	344304.986	344305.125(050)	344305.3(20)
6	1	1.5	1	0	0.5	0	344427.241	344427.362(050)	
7	2	1.5	2	1	0.5	1	687155.213		687157.17(17)
8	2	1.5	1	1	0.5	0	687169.251		687171.00(17)
9	2	2.5	3	1	1.5	2	687959.193		687959.54(19)
10	2	2.5	2	1	1.5	1	687972.501		687972.66(17)
11	3	2.5	3	2	2.5	3	1028194.809		1028202.5(10)
12	3	2.5	2	2	2.5	2	1028506.511		1028514.2(10)
13	3	3.5	4	2	2.5	3	1031102.404		1031104.29(21)
14	3	3.5	3	2	2.5	2	1031107.936		1031104.29(21)
15	4	3.5	4	3	3.5	4	1369778.585		1369797.0(10)
16	4	3.5	3	3	3.5	3	1370087.846		1370107.5(10)
17	4	3.5	4	3	2.5	3	1372686.180		1372700.06(98)
18	4	3.5	3	3	2.5	2	1372689.271		1372700.06(98)
19	4	4.5	5	3	3.5	4	1373479.987		1373485.81(55)
20	4	4.5	4	3	3.5	3	1373483.034		1373485.81(55)
21	6	5.5	6	5	4.5	5	2054132.512		2054170.48(71)
22	6	5.5	5	5	4.5	4	2054133.859		2054170.48(71)
23	6	6.5	7	5	5.5	6	2054924.631		2054944.05(82)
24	6	6.5	6	5	5.5	5	2054925.966		2054944.05(82)

a Five vibrational contracted basis functions |X ^2^Σ^+^, *v* = 0, 1, 2, 3, 4⟩ were used in this case. All frequencies are given in MHz.

Finally, we list two calculated *S* branch (*ΔJ* = 2) transitions in the second and fourth rows of Table 7. These hyperfine-induced transitions are much weaker than the two *R* branch (*ΔJ* = 1) transitions in the first and third rows.

**Table 7:** Comparison of the Line Positions and Strengths in the *R* and *S* Branches of ^24^Mg^1^H X ^2^Σ^+^, *v* = 0 Hyperfine Transitions^a^

no.	*F*′	τ′	*J*′	*F*″	τ″	*J*″	νDuo	ν_PG_	*S*Duo	*S*_PG_
1	2	+	2.5	1	–	1.5	687972.5015	687973.4786	1.7558441	1.7558510
2	2	+	2.5	1	–	0.5	689094.2323	689095.2094	0.0053314	0.0053315
3	3	–	3.5	2	+	2.5	1031107.9360	1031110.8777	2.8371019	2.8371270
4	3	–	3.5	2	+	1.5	1033046.9552	1033049.8969	0.0014804	0.0014805

a Line positions are given in MHz. Five vibrational contracted basis functions |X ^2^Σ^+^, *v* = 0, 1, 2, 3, 4⟩ were used in this case. The line strength, *S* [Debye^2^], has the same definition as that in PGOPHER when the intensity unit option of PGOPHER, IntensityUnit, is chosen as HonlLondon, and the transition dipole moment is set to 1 D.

## 7 Conclusion

We demonstrate an algorithm for the calculation of the hyperfine structure of diatomic molecules based on a variational treatment of nuclear motion. Nuclear magnetic dipole coupling terms including Fermi-contact, nuclear spin-electron spin dipole–dipole interaction, nuclear spin–orbit, nuclear spin–rotation, and nuclear electric quadrupole interaction terms are considered in our calculation. New modules for the hyperfine structure calculation are added to the flexible variational nuclear-motion package Duo.^21^

On the basis of the eigenfunctions and eigenvalues of ***J***, a parity-conserved rovibronic Hamiltonian matrix of particular total angular momentum, ***F***, is constructed and diagonalized. The hyperfine wave functions are finally represented using a Hund’s case (a_β_) basis set. Hyperfine-resolved line lists for diatomic molecules can be computed depending on the hyperfine energy levels and wave functions. To test the new module, we calculate the hyperfine structure of the *v* = 0, X ^2^Σ^+^ state of ^24^ MgH. The results of Duo and PGOPHER show excellent agreement for both line positions and line strengths. The Duo code and the input file used for ^14^N^16^O and ^24^MgH are available at https://github.com/ExoMol/Duo.

Our newly developed methodology builds a bridge between calculations of electronic motion and nucleus motion of diatomic molecules which makes it possible to calculate nuclear magnetic dipole and electric quadruple hyperfine structure effects from first principles. Some hyperfine coupling constants considered in this work may be calculated by quantum chemistry programs, for example, DALTON^50^ and CFOUR.^51^ It is also possible to evaluate them manually after obtaining electronic wavefunctions.^37^ We will discuss the *ab initio* calculation of hyperfine coupling constants in future work.

The current implementation only allows for nuclear spin effects on one atom and neglects coupling between electronic states. The hyperfine coupling between two electronic states is known to be important for some molecules. For instance, to analyze the spectrum of I^35^Cl, Slotterback et al. also included the hyperfine coupling terms between X ^1^Σ^+^ and A ^3^Π states.^52^ Implementing this effect in Duo would require some further work on the matrix elements but should not be a major undertaking. Treating the case where both atoms possess a nuclear spin introduces another source of angular momentum, and the interaction between the two nuclei also introduces new matrix elements.^12^ Here there are two possibilities, homonuclear systems, such as ^1^H_2_ or ^14^N_2_, can be treated by generalizing the scheme given in this paper. Heteronuclear systems, such as ^1^H^14^N, are a little more complicated as they give rise to different possible coupling schemes.^13^ Our plan is to gradually update Duo for each of these cases as the need arises.

## Supporting Information Available

Duo and PGOPHER input files used in this work (PDF)

## Notes

Duo is an open-source software, which is available at https://github.com/ExoMol/Duo, where Duo input files used in this work can be found.
