# Supplementary material for: A Method for the Variational Calculation of Hyperfine-Resolved Rovibronic Spectra of Diatomic Molecules
Source: J Chem Theory Comput. 2022 Feb 11;18(3):1808–20. doi: 10.1021/acs.jctc.1c01244 (PMC9097294; doi:10.1021/acs.jctc.1c01244)
Supplement: Supplementary file 1 — ct1c01244_si_001.pdf [file ct1c01244_si_001.pdf]

Supporting information

This file contains the supporting information of the paper titled: “A Method for the Variational Calculation of Hyperfine-Resolved Rovibronic Spectra of Diatomic Molecules”.

Qianwei Qu, Sergei N. Yurchenko, and Jonathan Tennyson*

*j.tennyson@ucl.ac.uk

Contents

Overview 2

DUO input file of MgH 3

DUO input file of NO.. 5

PGOPHER input file of MgH.. 8

PGOPHER input file of NO 9

Overview

DUO is a general open-source program for calculating spectra of diatomic molecules, which is available at https://github.com/ExoMol/Duo, where input files used in this work can be found.

The DUO and PGOPHER input files used in this work are also provided below.

DUO input file of MgH

```
atoms Mg-24 H-1

molecule MgH

(Total number of states taken into account)
nstates 1


(Total angular momentum quantum  - a value or an interval)
jrot 0.5 - 10.5

(Defining the integration grid)
grid
  npoints 401  (odd)
  range 1.0,5.0
  type 0   (nsub)
end

ASSIGN_V_BY_COUNT

(heteronuclear atom)
symmetry Cs(M)


(vmax for different states X-11 A-3 B-4)
CONTRACTION
  vmax      5 (or 1)
END


DIAGONALIZER
 SYEV   (SYEVR)
 nroots  600
end

poten 1
name 'X2Sigma+'
lambda 0
symmetry +
mult   2
type MLR_DS
    values
    V0           0.
    RE         1.7296850
    AE           11104.26
    RREF         2.73
    P            5
    Q            4
    N            12
    RHO          0.810
    B0           1.15492604850000e+00
    B1           1.04962030432982e+00
    B2           2.63821695711573e+00
    B3           2.01213050303404e+00
    B4          -6.09116777176108e-01
    B5           3.70028924733247e+00
    B6           2.02940905897105e+01
    B7           1.50140767755608e+01
```

```
    B8          -4.38643099381244e+01
    B9          -8.05444477366607e+01
    B10         -2.47901962028713e+01
    B11          3.04216704187525e+01
    B12          1.80314080042298e+01
    C1           0.
    C2           0.
    C3           0.
    C4           0.
    C5           0.
    C6     2.775e5
    C7           0.
    C8     3.454e6
    C9           0.
    C10           4.614e7
end


spin-rot  1 1
name "<X2Sigma+|SR|X2Sigma+>"
spin 0.5
lambda 0
factor 1.0
type polynom
values
    A0          0.0263785488559555 (cm-1)
end

hyperfine
I 0.5
end

hfcc-bf 1 1
name "<X2Sigma+|FC|X2Sigma+>" (Fermi-contact)
spin 0.5
lambda 0
factor 1.0
type polynom
values
    A0          1.026958234331E-02 (cm-1)
end


hfcc-c 1 1
name "<X2Sigma+|SDND_C|X2Sigma+>" (Electron spin - nuclear spin dipole-
dipole, c)
spin 0.5
lambda 0
factor 1.0
type polynom
values
    A0          1.598439144190E-04 (cm-1)
end


dipole  1 1
name "<X2Sigma+|DMZ|X2Sigma+>"
spin   0.5 0.5
lambda  0  0
factor   1   (0, 1 or i)
type polynom
values
    A0           1 (for test)
end
```

DUO input file of NO

```
atoms N-14 O-16

molecule NO

(Total number of states taken into account)
nstates 1

(Total angular momentum quantum  - a value or an interval)
jrot 0.5 - 100.5

solutionmethod Sinc

(Defining the integration grid)
grid
  npoints  701  (odd)
  range  0.6, 4.0
  type 0
end


symmetry Cs(M)


DIAGONALIZER
 SYEV   (SYEVR)
 enermax 53000.0
end


CONTRACTION
  vib
  vmax      1
END

poten 1
name "X2Pi"
lambda 1
mult   2
type   EMO
values
    VE           0.00000000000000E+00
    RE           0.11507863151853E+01
    DE           0.52495307750971E+05
    RREF        -0.10000000000000E+01
    PL           0.40000000000000E+01
    PR           0.40000000000000E+01
    NL           0.20000000000000E+01
    NR           0.10000000000000E+02
    B0           0.27657327621232E+01
    B1           0.17739962868001E+00
    B2           0.12996658564591E+00
    B3           0.18174776803043E+01
    B4          -0.97678608243932E+01
    B5           0.32552617956793E+02
    B6          -0.57640022462208E+02
    B7           0.55246373834427E+02
    B8          -0.21231743969255E+02
    B9           0.00000000000000E+00
    B10          0.00000000000000E+00
```

```
end


spin-orbit  1 1
name "<X2Pi|LSZ|X2Pi>"
spin   0.5 0.5
lambda  1  1
sigma  0.5 0.5
units  cm-1
factor    1.0   (0, 1 or i)
type polynom
values
    A0           60 (cm-1)
end

hyperfine
    I 1
end

hfcc-a 1 1
name "<X2Pi|NSO|X2Pi>" (Nuclear spin-orbit)
spin 0.5
factor 1.0
type polynom
values
    A0           0.1 (cm-1)
end

hfcc-bf 1 1
name "<X2Pi|FC|X2Pi>" (Fermi-contact)
spin 0.5
factor 1.0
type polynom
factor 1
values
    A0           0
end

hfcc-c 1 1
name "<X2Pi|SDND_C|X2Pi>" (Electron spin - nuclear spin dipole-dipole, c)
spin 0.5
factor 1.0
type polynom
values
    A0           0
end

hfcc-d 1 1
name "<X2Pi|SDND_D|X2Pi>" (Electron spin - nuclear spin dipole-dipole, d)
spin 0.5
factor 1.0
type polynom
values
    A0           0
end

hfcc-ci 1 1
name "<X2Pi|NSR|X2Pi>" (Nuclear spin - rotation)
spin 0.5
factor 1.0
type polynom
values
    A0           0
end
```

```
hfcc-eqq0 1 1
name "<X2Pi|eQq0|X2Pi>" (Electric quadrupole eQq0)
spin 0.5
factor 1.0
type polynom
values
    A0           0
end

hfcc-eqq2 1 1
name "<X2Pi|eQq2|X2Pi>" (Electric quadrupole eQq2)
spin 0.5
factor 1.0
type polynom
values
    A0           0
end


dipole  1 1
name "<X,2Pi|DMC|X,2Pi>"
spin   0.5 0.5
lambda  1  1
factor   1   (0, 1 or i)
type polynom
values
    A0           1 (for test)
end
```

PGOPHER input file of MgH


```
<?xml version="1.0"?>
<Mixture Units="MHz" Version="PGOPHER 10.1.182 4 Dec 2018 16:58 64 bit
Unicode (Delphi 32/32)" PlotUnits="MHz" IntensityUnits="HonlLondon">
  <Species Name="Species" Jmax="100">
    <LinearMolecule Name="LinearMolecule" nNuclei="1">
      <LinearManifold Name="Ground" Initial="True" LimitSearch="True"
Colour="Green" Jmin="1" Jmax="21">
        <Linear Name="v=0" S="1">
          <Parameter Name="B" Value="171976.1782"/>
          <Parameter Name="gamma" Value="790.809"/>
          <Parameter Name="D" Value="10.6212"/>
          <LinearNucleus Name="Nucleus1" Spin="1">
            <Parameter Name="b" Value="306.277"/>
            <Parameter Name="c" Value="4.792"/>
          </LinearNucleus>
        </Linear>
      </LinearManifold>
      <LinearManifold Name="Excited" LimitSearch="True" Colour="Maroon"
Jmin="1" Jmax="21">
        <Linear Name="v=0" S="1">
          <Parameter Name="B" Value="171976.1782"/>
          <Parameter Name="gamma" Value="790.809"/>
          <Parameter Name="D" Value="10.6212"/>
          <LinearNucleus Name="Nucleus1" Spin="1">
            <Parameter Name="b" Value="306.277"/>
            <Parameter Name="c" Value="4.792"/>
          </LinearNucleus>
        </Linear>
      </LinearManifold>
      <TransitionMoments Bra="Excited" Ket="Ground">
        <SphericalTransitionMoment Bra="v=0" Ket="v=0"/>
      </TransitionMoments>
    </LinearMolecule>
  </Species>
  <Parameter Name="Fmin" Value="-1428014.014336"/>
  <Parameter Name="Fmax" Value="1429112.48665472"/>
  <FormSettings Name="ConstantsWindow" ActiveObject="Linear: Species -
LinearMolecule - Excited - v=0"/>
  <FormSettings Name="LineWindow"
Lines="
LinearMolecule     Excited      2      1      1      Ground 1      0      1
      344305.116367102          .0037691334245824  0      rR1(0.5)2,1
      Excited v=0 1.5  1 F1e  2 - Ground v=0 0.5  0 F1e  1
LinearMolecule     Excited      1      1      2      Ground 0      0      1
      344427.371275493          .00121822773215854 0      rR1(0.5)1,0
      Excited v=0 1.5  1 F1e  1 - Ground v=0 0.5  0 F1e  0
LinearMolecule     Excited      1      1      1      Ground 1      0      1
      342997.766092412          .00121816584469268 0      rQ21(0.5)1,1
      Excited v=0 0.5  1 F2f  1 - Ground v=0 0.5  0 F1e  1
LinearMolecule     Excited      1      1      1      Ground 0      0      1
      343305.640424507          .0010433630068065  0      rQ21(0.5)1,0
      Excited v=0 0.5  1 F2f  1 - Ground v=0 0.5  0 F1e  0
LinearMolecule     Excited      1      1      2      Ground 1      0      1
      344119.496943398          .00104331000268357 0      rR1(0.5)1,1
      Excited v=0 1.5  1 F1e  1 - Ground v=0 0.5  0 F1e  1
LinearMolecule     Excited      0      1      1      Ground 1      0      1
      343117.465267905          .000753828788603465      0
      rQ21(0.5)0,1 Excited v=0 0.5  1 F2f  0 - Ground v=0 0.5  0 F1e  1
"/>
```

```
</Mixture>
```

PGOPHER input file of NO


```
<?xml version="1.0"?>
<Mixture Version="PGOPHER 10.1.182 4 Dec 2018 16:58 64 bit Unicode
(Delphi 32/32)" PlotUnits="MHz" IntensityUnits="HonlLondon">
  <Species Name="Species" Jmax="100">
    <LinearMolecule Name="LinearMolecule" nNuclei="1" Jmax="7">
      <LinearManifold Name="Ground" Initial="True" LimitSearch="True">
        <Linear Name="v=0" S="1" Lambda="Pi">
          <Parameter Name="B" Value="1.69608401191395"/>
          <Parameter Name="A" Value="120"/>
          <LinearNucleus Name="Nucleus1" Spin="2">
            <Parameter Name="a" Value="0.1"/>
          </LinearNucleus>
        </Linear>
      </LinearManifold>
      <LinearManifold Name="Excited" LimitSearch="True">
        <Linear Name="v=1" S="1" Lambda="Pi">
          <Parameter Name="B" Value="1.69608401191395"/>
          <Parameter Name="A" Value="120"/>
          <LinearNucleus Name="Nucleus1" Spin="2">
            <Parameter Name="a" Value="0.1"/>
          </LinearNucleus>
        </Linear>
      </LinearManifold>
      <TransitionMoments Bra="Excited" Ket="Ground">
        <SphericalTransitionMoment Bra="v=1" Ket="v=0"/>
      </TransitionMoments>
    </LinearMolecule>
  </Species>
  <Parameter Name="Fmin" Value="-4766308.19151973"/>
  <Parameter Name="Fmax" Value="4769974.58243629"/>
  <FormSettings Name="ConstantsWindow" ActiveObject="LinearNucleus:
Species - LinearMolecule - Excited - v=1 - Nucleus1"/>
</Mixture>
```
